# Type D personality and post-traumatic stress disorder symptoms among intensive care unit nurses: The mediating effect of resilience

**DOI:** 10.1371/journal.pone.0175067

**Published:** 2017-04-05

**Authors:** Geum-Jin Cho, Jiyeon Kang

**Affiliations:** 1 Neurological Intensive Care Unit, Dong-A University Medical Center, Busan, South Korea; 2 Department of Nursing, Dong-A University, Busan, South Korea; Stellenbosch University, SOUTH AFRICA

## Abstract

The purpose of this study was to investigate the relationship between Type D personality and post-traumatic stress disorder (PTSD) symptoms of intensive care unit (ICU) nurses and to determine the mediating effect of resilience on this relationship. A cross-sectional survey was performed with 179 ICU nurses from 7 hospitals in Gyeong-Nam province, South Korea. The Type D personality, resilience, and PTSD symptoms of subjects were measured using a self-report questionnaire. The mediating effect was analyzed by a series of hierarchical multiple regressions. A total of 38.6% of the study participants turned out to have Type D personality. The Type D personality was positively correlated with PTSD symptoms, and negatively correlated with resilience. There was a negative correlation between resilience and PTSD symptoms. The indirect effect of Type D personality on PTSD symptoms via resilience (β = .51, *p* < .001) was smaller than the direct effect (β = .58, *p* < .001). Based on the above results, it can be concluded that resilience had a partial mediating effect on the relationship between Type D personality and PTSD symptoms of ICU nurses. Further studies need to be done to develop interventions for enhancing resilience in ICU nurses.

## Introduction

Working in a stressful setting such as intensive care unit (ICU) may have detrimental effects on nurses’ psychological health. “Post mortem care, seeing patients die, involvement with end-of-life care, open surgical wounds, massive bleeding, trauma-related injuries, and performing cardiopulmonary resuscitation” are a few examples of traumatic events that ICU nurses confront at their workplace [1]. Some of these traumatic events such as witnessing the death or serious injury may cause post-traumatic stress disorder (PTSD) in ICU nurses.

PTSD is a psychological problem that develops in certain people who have experienced traumatic or life threatening events [2]. There has been much controversy about the definition and diagnostic criteria of PTSD. The scope of traumatic events in the fourth edition of Diagnostic and Statistical Manual of Mental Disorders (DSM-IV) was broad, but the newest edition, DSM-5 narrowed it to “actual or threatened death, serious injury, or sexual violence”. This limited definition of “trauma exposure” led to a substantial reduction in the incidence of PTSD [3]. Nevertheless, most ICU nurses are still witnessing those traumatic events of DSM-5 on a daily basis, and they may be at increased risk for developing PTSD symptoms. Indeed, 20–30% of ICU nurses had symptoms of PTSD, and 70% of those experienced PTSD symptoms for more than 3 months [1, 4]. PTSD symptoms directly impact the health of ICU nurses, and eventually influence outcomes for both the nursing workforce and patients [5].

Meanwhile, not all people who experience traumatic events will develop PTSD. The Type of symptoms and the severity of PTSD are expressed variably according to the victim’s subjective response to the traumatic event [6]. Specially, in situations of chronic stress, individual factors or vulnerability have a greater impact on the development of PTSD than the severity of the traumatic event itself [7]. One of the individual factors that affect PTSD is Type D personality [8]. The Type D personality consists of negative affectivity and social isolation, and is characterized by a depressive, anxious, and inappropriately worrying personality [9]. The proportion of Type D personality among clinical nurses is higher than that of general population, reaching at 36–38%, and nurses with Type D personality experience more compassion fatigue, burnout, and job stress than those with non-Type D personality [10, 11]. Previous studies have observed that Type D personality was positively correlated with PTSD in a group of violence victims [12] and firefighters [13].

Additional related factors linked to PTSD include resilience. Resilience is a psychological factor that mitigates the negative effects of trauma and promotes post-traumatic growth following the impacting events [14]. Luthar and colleagues [15] pointed out resilience as one of the most important factors that affects post-traumatic adaptation. As an individual characteristic, resilience refers to a positive ability to overcome stress or adversity and adapt successfully [16]. Resilience is also a dynamic concept that encompasses positive adaptation and individual growth in changing and challenging environments, and it can be created, maintained, or extinguished [17]. Because of these features, resilience has been the target of intervention to curtail the negative effects of PTSD. Several recent studies [18–20] have demonstrated the protective or mediating role of resilience on the relationship between predictive factors and PTSD symptoms among various populations.

ICU nurses experience more PTSD symptoms than general nurses [1], and resilient nurses utilize positive coping skills to overcome stressful events [21]. Until now, studies have examined the relationship between Type D personality and PTSD, and the relationship between resilience and PTSD. However, very few studies have examined the mediating role of resilience in relation to these variables. Accordingly, the purpose of the present study was to investigate the mediating effect of resilience on the relationship between Type D personality and PTSD symptoms in ICU nurses. The detailed objectives were: (1) to examine the degree of Type D personality, resilience, and PTSD symptoms of ICU nurses, (2) to examine the relationship between Type D personality, resilience, and PTSD symptoms of ICU nurses, and (3) to determine whether the relationship of Type D personality and PTSD symptoms was mediated by resilience.

## Methods

### Design

This study employed a cross-sectional survey to explain the relationships among Type D personality, resilience, and PTSD symptoms in ICU nurses.

### Sampling and data collection

The data were collected from August 1 to 31, 2013 using self-report questionnaires. A convenience sample of nurses was obtained from 7 hospitals in Gyeong-Nam province, South Korea. Selection criteria included nurses with at least 6 months clinical experience in ICUs, and participants had to understand the study aims and provide written informed consent.

Sample size was estimated using the G-power 3.1 program. For multiple regression analysis, the minimum required number of participants was 133, when considering a significance level (α) of .05, an effect size (f^2^) of .18 [22], test power (1-β) of .90, and the number of predicting variables being 12. Considering potential dropouts, questionnaires were sent to 235 ICU nurses, of which 219 were returned. After excluding 40 questionnaires (21 were not completed, 19 had the same answers marked for all questions), 179 questionnaires were used in the final analysis.

### Measurements

#### Type D personality

The Korean version of the Type D scale-14, DS14 [23], originally developed by Denollet [9] was used to measure Type D personality. The DS14 consists of 14 items in 2 subscales: 7 items on negative affectivity and 7 items on social inhibition. Respondents answer on a 5-point Likert scale that ranges from 0 (*false*) to 4 (*true*). Both subscales can be calculated as continuous variables (minimum 0 to maximum 28). Respondents can be categorized as Type D when they score ≥10 on both scales [9]. Both a categorical approach and dimensional scores were utilized in this study. The internal consistency (Cronbach's α) values of the questionnaire reported by Denollet [9] were .88 and .86, respectively, for negative affectivity and social inhibition, and those for the current study were .90 and .92.

#### Post-traumatic stress disorder symptoms

PTSD symptoms were measured using the Korean version of the Posttraumatic Diagnostic Scale (PDS) [23], originally developed by Foa et al. [24]. We utilized 17 items from part 3 of the PDS that describes DSM-IV diagnostic criteria and symptom severity of PTSD: 5 items for re-experiencing, 7 items for avoidance, and 5 items for arousal symptoms. Respondents answer each item on a 4-point scale (0 = *not at all* to 3 = *more than 5 times in a week*) over a period concerning the past month; thus higher scores indicate that the respondent experienced more PTSD symptoms. If a respondent scores ≥20, he or she could be categorized into the high-risk group. Ahn [25] reported the Cronbach’s α of the Korean version of the PDS to be .91, whereas it was .90 in our study.

#### Resilience

Resilience was measured using the Korean version of the Connor-Davidson Resilience scale [26], originally developed and validated by Connor and Davidson [27]. This scale consists of 25 items with a total score from 0 to 100. Responses were made on a Likert scale ranging from 0 (*Not at all*) to 5 (*Very much*). Higher scores indicate more resilience. The Cronbach's α of the questionnaire reported by Connor and Davidson [27] was .89, Cronbach's α of the Korean version was .91 [26], and Cronbach’s α = .92 for the present study.

### Data analysis

The collected data were analyzed using IBM SPSS for Windows 22.0 (IBM Corporation, Armonk, NY, USA). Descriptive statistics were employed to assess participants’ characteristics. The differences in PTSD symptoms according to participants’ characteristics were analyzed by t-tests and ANOVA. Correlations between variables were analyzed using Pearson’s correlation coefficients. To test the mediating effect, a series of hierarchical multiple regressions was performed according to Baron and Kenny’s [28] procedures and Sobel test.

### Ethical consideration

The Institutional Review Board of Dong-A university approved this study (Approval number: 13–071). Approval from the relevant institution directors where data collection took place was also obtained. Finally, all participants provided written informed consent prior to completing the questionnaires.

## Results

### Participants’ characteristics and PTSD symptoms

A total of 179 ICU nurses participated. Most of them were female (93.3%), were general staff nurses (91.9%), were unmarried (78.2%), had a bachelor degree (73.7%), and worked for a tertiary hospital (64.3%). Their mean age was 28.80±5.95 years, and clinical experience was 54.12±47.30 months on average. In terms of ICU Type, 33.5% worked in a mixed ICU, 20.7% for a neurological ICU, and 17.7% each for a medical and surgical ICU. There were no statistically significant differences in PTSD symptoms according to participants’ characteristics (Table 1).

**Table 1:** Study Participants’ Characteristics and PTSD Symptoms (N = 179).

Characteristics Categories		n (%)	PTSD symptoms		
			M±SD	t/F	*p*-value
Gender	Male	12 (6.7)	9.25±7.05	1.44	0.151
	Female	167 (93.3)	12.65±7.93		
Age(years)	20–24	51 (28.5)	10.53±6.61	2.90	0.057
	25–29	60 (33.5)	14.12±8.42		
	≥4.	68 (38.0)	12.34±8.11		
	M±SD	28.80±5.95			
Marital status	Married	39 (21.8)	11.08±7.69	1.20	0.232
	Unmarried	140 (78.2)	12.79±7.95		
Religion	Yes	99 (55.3)	12.55±8.02	0.24	0.813
	No	80 (44.7)	12.26±7.80		
Education	Associate	47 (26.3)	11.13±6.84	1.31	0.193
	≥ Bachelor	132 (73.7)	12.88±8.23		
Type of	Secondary	64 (35.8)	11.59±8.12	1.04	0.299
hospital	Tertiary	115 (64.2)	12.88±7.78		
ICU Type	Mixed	60 (33.5)	11.70±8.30	2.35	0.056
	Surgical	32 (17.9)	11.84±6.12		
	Medical	31 (17.3)	13.26±6.67		
	Neurological	36 (20.1)	10.86±7.89		
	Cardiac	20 (11.2)	17.00±9.77		
Position	Staff nurse	166 (92.7)	12.60±8.00	1.11	0.269
	≥ Charge nurse	13 (7.3)	10.08±6.36		
Clinical	≤li	28 (15.6)	10.93±6.31	0.68	0.639
experience	13–24	23 (12.8)	11.61±9.07		
(months)	25–36	17 (9.5)	15.18±9.17		
	37–60	33 (18.4)	12.45±6.96		
	61–120	44 (24.6)	12.82±8.28		
	≥2.8	34 (19.0)	12.26±8.14		
	M±SD	54.12±47.30			

### Type D personality, resilience, and PTSD symptoms

Descriptive statistics (means, standard deviations) of the main variables are as followed: Type D personality 19.44±11.99, resilience 59.56±12.32, PTSD symptoms 12.42±7.91. Thirty-eight point six percent of study participants were classified as having a Type D personality, and 18.2% were classified as a high-risk group for PTSD.

Correlation analysis showed that Type D personality was positively correlated with PTSD symptoms (r = .58, p < .001), and negatively correlated with resilience (r = -.43, p < .001). Meanwhile, resilience was negatively correlated with PTSD symptoms (r = -.39, p < .001; Table 2).

**Table 2:** Descriptive Statistics and Correlations between Variables (N = 179).

Variables	M±SD	1	2	3
1. PTSD symptoms	12.42±7.91	-		
2. Type D personality	19.44±11.99	0.58 (< .001)	-	
3. Resilience	59.56±12.32	-0.39 (< .001)	-0.43 (< .001)	-

### Mediating effect of resilience

Prior to testing the mediating effect, we confirmed the preconditions of the data for regression analysis. There was no multicollinearity problem for the regression model because the variance inflation factors of all independent variables were less than 10. A residual analysis using the Durbin-Watson test showed that there was no autocorrelation. Since no participants’ characteristic was significantly associated to PTSD symptoms, we did not enter any confounding variables into the regression models.

**Fig 1:**
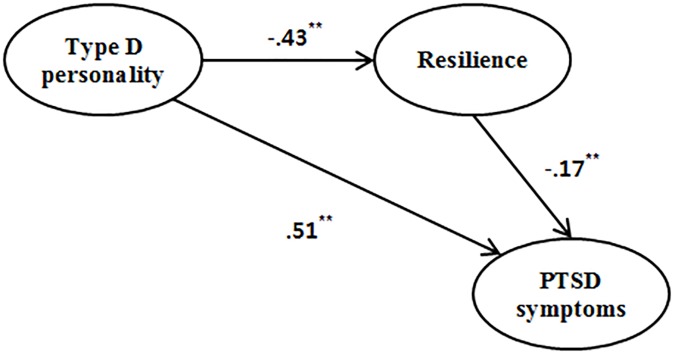
Model for Mediating Effect of Resilience. Note: **p < .001.

To determine the mediating effect of resilience on the relationship between Type D personality and PTSD symptoms, the 4-step mediation analysis proposed by Baron and Kenny [28] was performed as follows:

· Step 1 regression analysis showed that Type D significantly predicted PTSD symptoms (*β* = .58, *t* = 9.43, *p* < .001).
· Step 2 regression analysis showed that Type D significantly predicted resilience (*β* = -.43, *t* = -6.33, *p* < .001).
· Step 3 regression analysis (both Type D and resilience as predictors) showed that resilience significantly predicted PTSD symptoms (*β* = .51, *t* = -2.55, *p* < .001).
· Step 4 when resilience was entered into the equation between Type D and PTSD symptoms, the *β* weight for Type D was reduced. As the relationship between Type D and PTSD symptoms was not reduced to non-significance, it was revealed that resilience partially mediated the relationship between Type D personality and PTSD symptoms (Table 3 and Fig 1). The results of the Sobel test also verified that the mediating effect of resilience was significant (z = 2.37, *p* = .018).

**Table 3:** Results of Hierarchical Regressions for Medicating Effects (*N* = 179).

Paths	SE	Standardized β	t	p-value
Type D → PTSD	0.04	.58	9.43	< .001
Type D → Resilience	0.07	-.43	-6.33	< .001
Resilience → PTSD	0.04	-.17	-2.55	< .001
Type D and Resilience → PTSD	0.04	.51	7.55	< .001

## Discussion

Among the ICU nurses in the current study, 18.2% could be considered as high risk group of PTSD. This is similar to the results of previous studies [4, 29] in which 18–21% of ICU nurses were observed to meet diagnostic criteria for PTSD. ICU nurses develop PTSD symptoms from daily encounters with traumatic events such as dealing with severely injured or dying patients, involvement with end-of-life care, and performing cardiopulmonary resuscitation [1, 21]. If the symptoms of PTSD are not managed in the early stages, they may cause depression, anxiety, substance abuse, physical symptoms, and burnout syndrome [4, 30], as well. Therefore, it is necessary to prepare a measure for earlier assessment and appropriate management of PTSD symptoms in ICU nurses.

The prevalence of Type D personality was reported at 16.6–29.0% in the general population [31]. However, 38.3% of Turkish physicians and nurses [11] and 23–36% of Belgian nurses [22] were reported to have a Type D personality. In terms of prevalence, our study participants (38.6%) are on the higher side when compared to the general population, and are similar to medical personnel. According to Kim et al., the prevalence of Type D personality among nurses with more than 5 years of clinical experience increased up to 41%. They explained the cause of this high prevalence might be changes in personality due to repetitive exposure to work stress [10]. Karlsson et al. [32] have shown that a cognitive-behavioral intervention lowered the type D score of patients with coronary artery disease. The results of these two studies imply the possible variability of type D personality, and this speculation is not consistent with the assumption that type D personality is relatively stable psychological state [9]. The possibility of an alteration in type D personality needs to be confirmed by future prospective studies.

The current study has revealed that Type D personality is associated with PTSD symptoms in ICU nurses. Specifically, ICU nurses with Type D personality had experienced more PTSD symptoms. This is consistent with the results of a systematic review [31] that indicated the presence of a Type D personality had a negative impact on mental health status including PTSD. Kunst et al. [12] also observed Type D personality in violence victims predicted their PTSD. Type D personality is characterized by a combination of negative affectivity and social inhibition. An individual with negative affectivity has a tendency to experience distress, anxiety, and worrying emotions, while an individual with social inhibitory characteristic tends to have a limited social network and to be uncomfortable in relating to others [33]. Accordingly, a Type D person is more likely to be vulnerable to stressful events, and is less likely to seek help or support from outside than a non-Type D [34, 35].

The above studies have all suggested an association between Type D personality and PTSD symptoms, but the current study is the first that shows this relationship among ICU nurses. We built and tested a regression model under the assumption that Type D personality was a predictive factor of PTSD symptoms. However, according to a qualitative study with ICU nurses [21], nurses who were experiencing PTSD had trouble forgetting negative experiences and emotions from patient care, and this affected their daily lives and interpersonal relationships. These features of PTSD outcomes are similar to Type D personality. Thus, we should be careful about coming to a conclusion on the causal relationship between these two variables with this cross-sectional study. Further research is needed to establish the precise direction of the relationship between Type D personality and PTSD symptoms.

The resilience of ICU nurses partially mediated the relationship between Type D personality and PTSD symptoms in the current study. In other words, PTSD symptoms can be improved by intervening resilience. This result supports Mealer and colleagues’ study [21] that observed that the higher the resilience in ICU nurses, the fewer PTSD symptoms that they developed. Considering the stable nature of the Type D construct, researchers have focused on the individuals’ adaptive ability to ameliorate the negative effects of Type D personality [31, 35, 36]. A resilient person experiences fewer negative psychological processes in response to the same traumatic events [14]. In addition, resilience is not an innate characteristics, it is a stable trajectory, a conscious effort, a capacity or a process that can be changed by training or education. The determinants of resilience vary depending on the situation, but a resilient individual can use various coping measures flexibly for specific situations [37]. Horn and colleagues [38] pointed out that resilience to PTSD was related to cognitive reappraisal, social support, active coping strategies, and the use of humor. More specifically, they proposed interventions such as mindfulness, meditation, or yoga for being resilient to PTSD. In line with this study, Mealer and colleagues [39] reported the feasibility and acceptability of a multimodal resilience training program developed for reducing PTSD symptoms of ICU nurses. This program consisted of education, consultation, meditation, and expressive writing, and interestingly, PTSD symptoms were decreased in both experimental and control groups. Thus, more rigorous clinical trials should be attempted to examine the effects of resilience training on reducing PTSD symptoms in ICU nurses.

The significance of the current study is its confirmation of the mediating effect of resilience on ICU nurses’ PTSD symptoms. Considering the high prevalence of Type D personality and PTSD, efforts to increase ICU nurses’ resilience are needed to mitigate the negative outcomes of PTSD. Of course, the present study has some limitations. First, we only assessed PTSD symptoms from ICU nurses, and did not assess the criterion A for PTSD. In addition, this study included only self-reported questionnaires. These can limit the suitability of the study results. Second, because the data were collected from nurses in a certain area of South Korea, the generalizability of the study findings may be limited. Finally, since this study adopted a cross-sectional design, we cannot confirm the direction of the causal relationship between the study variables. A prospective longitudinal study is needed to clarify the relationship between variables.

## Conclusions

We observed that the prevalence of Type D personality among ICU nurse was high at 38.6%, and ICU nurses with Type D personality tended to experience more PTSD symptoms. In addition, resilience in ICU nurses partially mediated the relationship between Type D personality and PTSD symptoms. Based on these findings, there is a need to conduct a prospective study in order to verify the causal relationship between Type D personality and PTSD symptoms. In addition, we suggest the intervention research and institutional policy development to enhance ICU nurses’ resilience to mitigate the negative effects of PTSD.

## Supporting information

S1 Dataset Data file. (XLSX)
